# Heavy Metal Accumulation and Human Health Risk in Mediterranean Mussels from the Southern Marmara Sea, Türkiye

**DOI:** 10.3390/toxics13121084

**Published:** 2025-12-17

**Authors:** Saadet Hacısalihoğlu

**Affiliations:** Department of Environmental Engineering, Faculty of Engineering and Natural Sciences, Bursa Technical University, Mimar Sinan Campus, Bursa 16310, Turkey; saadet.hacisalihoglu@btu.edu.tr

**Keywords:** Bursa, heavy metals, health risks assessment, Marmara Sea, *Mytilus galloprovincialis*, toxics

## Abstract

This study evaluated the accumulation of heavy metals (As, Cd, Cu, Hg, Pb, and Zn) in Mediterranean mussels (*Mytilus galloprovincialis*) collected from five coastal stations (Küçükkumla, Kurşunlu, Güzelyalı, Mudanya, and Zeytinbağı; *n* = 20 mussels per station; composited into one sample per site) along the southern coast of the Marmara Sea (Bursa, Türkiye), and assessed the associated potential health risks. Analyses using ICP-OES revealed spatial variations in metal concentrations among stations. Statistical analyses (*p* < 0.05) showed no significant differences in As levels, whereas Cd, Cu, Hg, Pb, and Zn concentrations differed significantly. Mudanya exhibited the highest levels of Zn and Hg, while Cd was particularly elevated in Mudanya and Güzelyalı. Kurşunlu showed the highest Cu concentrations, and Küçükkumla had the highest Pb levels. Human health risk assessments for the adult population (EDI, EWI, THQ, HI) were all below 1.0, indicating no appreciable non-carcinogenic risk under the assumed adult dietary exposure scenario, based on internationally recognized toxicological reference values (FAO/WHO, JECFA, and EC Regulation 1881/2006). However, relatively higher HI values in Mudanya (0.695) and Küçükkumla (0.646) suggest the need for closer monitoring. Overall, the findings demonstrate that mussels serve as effective bioindicators of coastal metal contamination in the southern Marmara Sea and underscore the importance of continuous biomonitoring to safeguard both ecosystem and public health.

## 1. Introduction

In recent years, the relationship between nutrition and public health has drawn increasing attention due to the growing prevalence of food-related illnesses [1,2]. Among these concerns, heavy metal contamination in seafood has emerged as a critical issue, particularly in coastal regions where anthropogenic pressures are high [3]. Given that seafood consumption represents an important exposure pathway for coastal populations, understanding metal accumulation in edible marine organisms is essential for public health protection.

Heavy metal pollution in marine environments represents a major global concern. The main sources of this contamination include industrial discharges, domestic wastewater, mining activities, agricultural runoff, maritime transport, and atmospheric deposition [4]. Once introduced into the environment, heavy metals persist for long periods due to their non-degradable nature, thereby undergoing bioaccumulation and biomagnification within the food web [5]. This not only poses a threat to the health of aquatic organisms but also creates serious toxicological risks for humans consuming contaminated seafood [6]. This issue is particularly relevant for semi-enclosed basins such as the Marmara Sea, where restricted water circulation can intensify pollutant retention and biological exposure.

Among edible marine organisms, mussels are among the most widely consumed seafood worldwide due to their high nutritional value and relatively low cost [3]. As filter-feeding organisms, mussels accumulate contaminants directly from their surrounding aquatic environment [7]. This biological feature makes them excellent bioindicators for monitoring marine pollution [8,9,10]. However, this same characteristic increases their potential to accumulate toxic metals such as lead (Pb), cadmium (Cd), mercury (Hg), and arsenic (As), which may pose health risks when mussels are consumed regularly over long periods [11,12].

Studies have shown that the levels of heavy metals in mussels can vary depending on several factors, including water quality, geographical location, industrial and urban activities, and seasonal changes [13,14]. Chronic exposure to these metals through food intake may lead to a variety of health issues, ranging from neurological damage and kidney dysfunction to carcinogenic effects [15]. Given the potential long-term risks associated with heavy metal ingestion, regular monitoring and comparison with internationally recognized regulatory limits have become essential. Despite the increasing number of studies on heavy metals in bivalves, there remains a limited understanding of spatial variability along the southern Marmara Sea and how these variations translate into human exposure risks. This gap underscores the need for integrated assessments combining bioaccumulation patterns with human health risk metrics [16].

The Marmara Sea is a significant region in terms of biodiversity, serving both export and local markets. Marine life in this area is influenced by various environmental factors, including water temperature, salinity, and pollution levels, all of which directly impact the diversity of marine products [17,18]. However, due to anthropogenic pressures such as rapid population growth, industrial expansion, and agricultural activities, this valuable ecosystem is increasingly threatened by a range of pollutants. Recent studies [18,19,20,21] conducted in the Marmara Sea have revealed that the region is severely affected by toxic pollution and mucilage contamination. In light of these findings, monitoring mussels from this region is critical due to their high filtration capacity and susceptibility to accumulating contaminants.

Accordingly, this study aimed to quantify the accumulation of six heavy metals (As, Cd, Cu, Hg, Pb, and Zn) in Mediterranean mussels collected from five coastal sites along the southern Marmara Sea and to assess potential human health risks associated with their consumption. We hypothesized that mussels from areas subject to higher anthropogenic pressure (particularly port zones and densely populated coastal districts) would exhibit elevated metal concentrations compared to less-impacted sites. This study addresses an important regional knowledge gap by integrating spatial bioaccumulation data with human health risk assessment, thereby contributing to ongoing pollution monitoring efforts and supporting public health protection strategies.

## 2. Materials and Methods

### 2.1. Study Area and Field Sampling

The Marmara Sea is a small inland sea situated in northwestern Türkiye, serving as a natural connection between the Black Sea and the Aegean Sea, and separating the Asian and European parts of the country. It spans approximately 280 km in length and 80 km in width, with a surface area of around 11,500 km^2^. The sea is bordered by major cities such as Istanbul, Bursa, İzmit, and Tekirdağ, which are characterized by high population densities and intensive industrial activities. The city of Bursa, located in the southeastern part of the Sea of Marmara, is the fourth most populous city in Türkiye and represents a significant industrial and commercial hub [22].

In this study, sampling locations were selected based on their proximity to sources of domestic, industrial, and agricultural pollution, as well as population density and economic activity. Geographical distance was not included as a statistical variable because the objective was to compare site-specific anthropogenic influences rather than spatial gradients. Each site was treated as an independent sampling unit, representative of its local environmental conditions. The sites were located within a ~60 km stretch of coastline, reflecting distinct levels of anthropogenic pressure.

**Figure 1:**
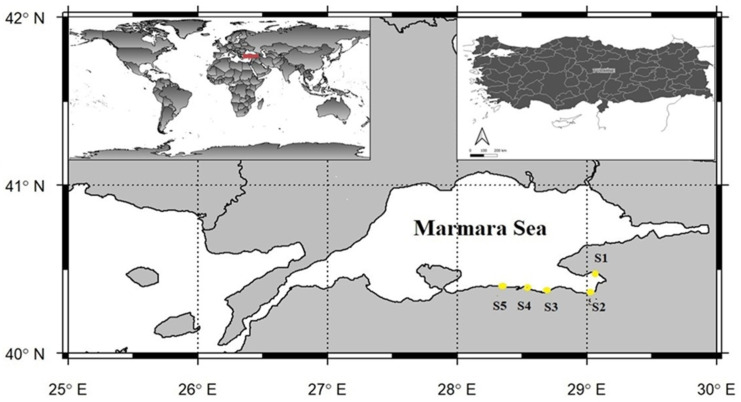
Study area of sampling points in the southern Marmara Sea. The red color represents Turkey’s location on the world map.

Sampling was limited to five designated stations (S1–S5) located within the southern part of the Sea of Marmara, within the administrative boundaries of Bursa Province (Figure 1). The characteristics of the sampling stations were defined as follows: S1 (Küçükkumla), a recreational marina area; S2 (Kurşunlu), a semi-urban site influenced by moderate human activity; S3 (Güzelyalı), a residential coastal zone; S4 (Mudanya), a port and shipyard area with intensive maritime operations; and S5 (Zeytinbağı), a rural coastal site with minimal anthropogenic influence.

The Mediterranean mussel (*Mytilus galloprovincialis*), a widely distributed species in the Sea of Marmara, was selected due to its high filtration capacity and ability to reflect local contamination levels. Sampling was conducted within a single season (August 2023) to minimize the effects of seasonal variability on metal accumulation, as temperature, salinity, and food availability can influence metal uptake in bivalves. The single-season sampling design is acknowledged as a limitation because it does not capture potential temporal fluctuations in metal accumulation.

Mussel samples were manually collected from coastal rocks at depths of 1–3 m at five different sampling sites (S1–S5) using stainless steel tongs and placed in pre-cleaned polyethylene containers. All sampling equipment was acid-washed with 10% HNO_3_ prior to use to prevent contamination. At each sampling site, 20 mussels (*n* = 20) were collected and depurated in aerated, filtered seawater for 12 h to remove gut contents, as recommended for accurate metal analysis. Mussels were selected to have similar shell lengths (8.0–10.0 cm); however, natural morphological variability resulted in slightly higher mean values (9.75 ± 2.27 cm). This deviation is attributed to natural size heterogeneity in field populations and does not affect compositing procedures or analytical outcomes.

Collected individuals were combined to create one composite sample per station, following established biomonitoring protocols [23,24]. Because composite sampling was used, biological variability within each site could not be statistically compared; therefore, all triplicate measurements represent analytical rather than biological replicates. Each composite sample was analyzed in triplicate to ensure analytical reproducibility.

### 2.2. Heavy Metal Analysis

Prior to analysis, the mussel samples were thawed at room temperature and thoroughly rinsed with ultrapure deionized water [21]. A total of 100 mussels were processed, and their soft tissues were homogenized for metal analysis. As filter-feeding organisms, mussels can accumulate both dissolved and particulate-bound contaminants, providing an indication of local metal bioavailability; however, their tissue concentrations reflect the bioavailable fraction rather than total environmental metal levels, which is an important consideration when interpreting results.

Heavy metal concentrations were analyzed using a VARIAN VISTA-MPX model (Palo Alto, California, ABD) Inductively Coupled Plasma-Optical Emission Spectrometer (ICP-OES). From each homogenized composite sample, 0.5 g of tissue was weighed into heat-resistant glass vessels for wet digestion. In this process, 65% nitric acid (HNO_3_) was added, mixed, and allowed to react for 30 min. The mixture was then heated at 100 °C for one hour and cooled. Following this, 65% perchloric acid (HClO_4_) [25,26,27,28,29] was added, and the same heating procedure was repeated. Because HClO_4_ is a highly reactive oxidizing agent, all digestions were performed in a certified perchloric acid fume hood to ensure laboratory safety.

For the ICP-OES analysis of arsenic (As), cadmium (Cd), copper (Cu), lead (Pb), and zinc (Zn), the digested samples were diluted with distilled water. Mercury (Hg) was prepared separately using 10% hydrochloric acid (HCl; Merck, Darmstadt, Germany) [30,31].

All measurements were performed in triplicate to ensure analytical precision; however, these triplicates represent analytical replicates, as each sampling station was represented by a single composite biological sample. Calibration curves were constructed using multi-element standard solutions (Merck, Germany), and all elements showed correlation coefficients (R^2^) greater than 0.999. Limits of detection (LOD) and limits of quantification (LOQ) were calculated as 3σ and 10σ of the blank signals, respectively. The LOD/LOQ values (mg/kg, wet weight) were as follows: As (0.012/0.040), Cd (0.001/0.004), Cu (0.003/0.010), Hg (0.002/0.007), Pb (0.005/0.017), and Zn (0.010/0.033). Certified reference material for bivalve tissue was not available at the time of analysis; therefore, method accuracy was verified using spike recovery tests, which yielded acceptable recoveries (92.4–104.4%). Given that recoveries ranged from 92.4% to 104.4%, potential analytical bias is considered minimal [32,33]. These results confirm that the applied analytical method provided acceptable precision, accuracy, and reproducibility for all metals. The operating conditions of the ICP-OES instrument were optimized according to manufacturer guidelines, including an RF power of 1300 W, plasma gas flow of 15 L/min, auxiliary gas flow of 1.5 L/min, and nebulizer flow of 0.75 L/min.

### 2.3. Data Analysis

Statistical analyses were performed using IBM SPSS Statistics version 28.0. Descriptive statistics, including mean values and standard deviations (SD), were calculated to summarize the concentrations of heavy metals across sampling sites. Prior to inferential testing, data distribution and variance homogeneity were evaluated using the Shapiro–Wilk normality test and Levene’s test, respectively. Only parameters that met the assumptions of normality and homogeneity were evaluated with one-way ANOVA. When ANOVA indicated statistically significant differences among stations (*p* < 0.05), Tukey’s HSD test was applied for post hoc pairwise comparisons. Pearson correlation analysis was used to examine potential relationships among metal concentrations, and correlation significance was evaluated at *p* < 0.05. Because each station was represented by a composite biological sample, statistical differences reflect analytical precision rather than biological variability, and thus site-level patterns should be interpreted with caution.

For human health risk assessment, Estimated Daily Intake (EDI), Estimated Weekly Intake (EWI), and Provisional Tolerable Weekly Intake (PTWI) values were calculated. PTWI values established by JECFA indicate the maximum tolerable weekly exposure without appreciable health risk; however, PTWI differs from the oral reference dose (RfD), which represents a contaminant-specific daily exposure threshold. Therefore, PTWI and RfD were applied according to their intended toxicological frameworks. Dietary exposure was considered acceptable when EWI < PTWI [34,35]. To evaluate non-carcinogenic risks, the Target Hazard Quotient (THQ) and Hazard Index (HI) were computed. THQ estimates the risk associated with exposure to a single metal, whereas HI represents the sum of all THQs and thus the cumulative risk from multiple contaminants. A THQ < 1.0 or HI < 1.0 indicates no appreciable health risk, while values > 1.0 may indicate potential concern [9,10,16]. Risk assessment was performed for two population groups—adults (70 kg body weight) and children (20 kg)—because exposure varies substantially with age and body mass. Detailed THQ and HI values for the child population are provided in Supplementary Table S2. The daily seafood consumption rate (CR) was obtained from the most recent Türkiye National Dietary Intake Survey, representing average national consumption. Exposure duration (ED) and exposure frequency (EF) followed US EPA standard assumptions. The formulas used for exposure and risk calculations are presented in Table 1.

## 3. Results

### 3.1. Heavy Metal Concentrations in the Mussels

**Figure 2:**
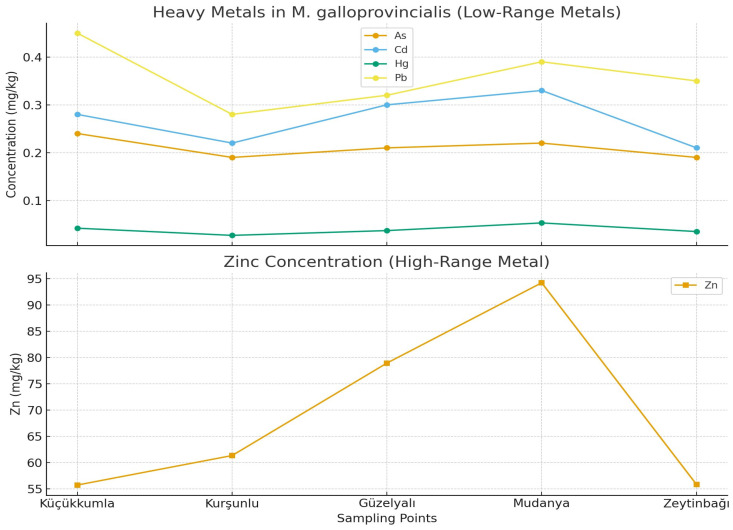
Mean concentrations of heavy metals (mg/kg, wet weight) in *M. galloprovincialis* collected from different sampling points.

In this study, concentrations of As, Cd, Cu, Hg, Pb, and Zn were measured in *M. galloprovincialis* collected from five coastal locations along the southern Marmara Sea. Descriptive statistics (mean ± SD) are provided in Table 2, and spatial patterns are shown in Figure 2. Among the analyzed metals, Zn displayed the highest concentrations across all stations, reaching its maximum value in Mudanya (94.25 mg/kg). In contrast, As showed the narrowest range (0.19–0.24 mg/kg), indicating relatively uniform distribution among sites. Cu concentrations varied between 1.79 and 3.11 mg/kg, with the highest levels observed in Kurşunlu.

Because each site was represented by a composite biological sample, the reported SD values reflect analytical precision rather than true biological variability. Therefore, spatial differences should be interpreted as site-level patterns, not within-site heterogeneity.

Because analytical replicates were used, statistical significance reflects analytical precision rather than biological variability; therefore, spatial comparisons should be interpreted as site-level patterns. One-way ANOVA results (Table 3) revealed statistically significant spatial differences for Cd, Cu, Hg, Pb, and Zn (*p* < 0.05), whereas As did not vary significantly among stations. Tukey’s HSD post hoc tests indicated that Mudanya had significantly higher Zn and Hg concentrations than other stations, and Kurşunlu exhibited significantly higher Cu concentrations. These findings align with known anthropogenic influences in these regions, including port activity, maritime operations, and semi-urban inputs.

**Figure 3:**
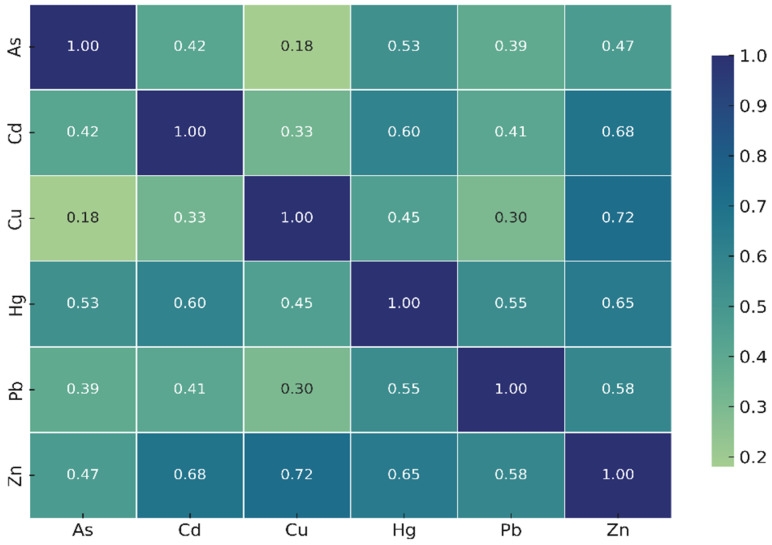
Pearson correlation heatmap (r values) of heavy metals in *M. galloprovincialis* from the southern Marmara Sea.

Pearson correlation analysis (Figure 3) revealed noteworthy positive correlations: Zn–Cu (shared metabolic or uptake pathways), Cd–Zn, and Cd–Hg. These correlations may suggest common accumulation mechanisms or shared environmental sources, although further environmental data (e.g., sediment, water concentrations) would be required to confirm source apportionment.

Positive correlations observed between Zn–Cu, Cd–Zn, and Cd–Hg may reflect shared physiological accumulation pathways or co-occurrence of these metals in the surrounding environment. Because each station was represented by a composite mussel sample, the correlations represent analytical associations rather than individual-level biological variation. As no parallel measurements of water or sediment were conducted, these correlations should not be interpreted as definitive evidence of common pollution sources; instead, they highlight patterns that warrant further investigation in future multi-matrix monitoring studies.

### 3.2. Food Safety Assessment and Regulatory Comparison

The measured concentrations of As, Cd, Cu, Hg, Pb, and Zn were compared with internationally recognized food safety limits, and the results are summarized in Table 4. Arsenic and cadmium concentrations were well below the maximum levels established by FAO/WHO and the European Commission, respectively. Similarly, mercury and copper values remained far below regulatory thresholds, indicating minimal concern for these metals.

In contrast, lead concentrations slightly exceeded the EU limit (0.30 mg/kg wet weight) at some stations, warranting attention given Pb’s well-known neurotoxic and hematological effects. Zinc concentrations also exceeded the general food guideline value (50 mg/kg) at all stations; however, Zn is an essential trace element, and its elevated levels in mussels do not directly imply toxicity. As discussed in the literature, Mediterranean mussels naturally exhibit high Zn levels, and when converted to wet-weight basis, they constitute only a modest component of daily dietary Zn intake. These factors should be considered when interpreting Zn exceedances in the context of food safety.

**Figure 4:**
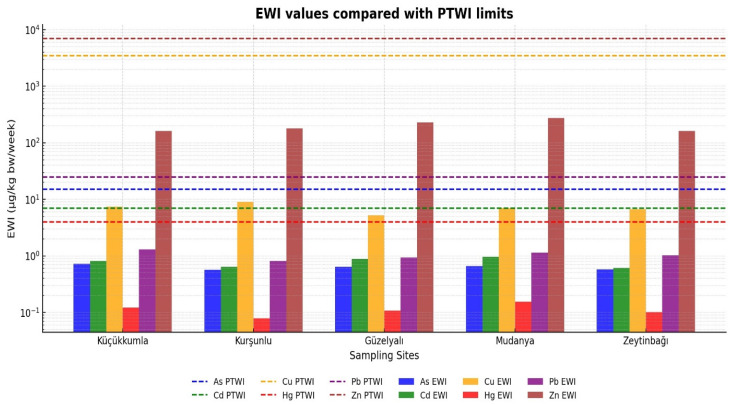
Estimated Weekly Intake (EWI) of heavy metals in *M. galloprovincialis* compared with their respective Provisional Tolerable Weekly Intake (PTWI) limits established by FAO/WHO–JECFA. Dashed horizontal lines indicate PTWI values for each metal, color-coded to match the bars.

Zinc values exceeded the general food guideline due to the naturally elevated Zn content characteristic of Mediterranean mussels; however, this does not directly imply a toxicological risk without considering actual dietary intake levels. To further evaluate potential health impacts, Estimated Weekly Intake (EWI) values were calculated and compared with Provisional Tolerable Weekly Intake (PTWI) limits (Figure 4). For all metals and all stations, EWI values remained below their respective PTWI thresholds, indicating no appreciable risk from weekly mussel consumption under the assumed exposure scenario. Zn and Cu contributed the highest proportions to weekly intake due to their comparatively higher concentrations, whereas Hg and As contributed minimally. A logarithmic y-axis was used in Figure 4 to enhance comparability among metals with different concentration magnitudes.

In Figure 4, dashed horizontal lines represent PTWI thresholds for each metal. A logarithmic y-axis is used to accommodate the wide concentration differences among metals and to improve visual comparability. Across all stations, EWI values remained below PTWI limits, indicating no appreciable health risk under the assumed consumption scenario. Although Zn and Cu exhibited the highest weekly intake contributions, their values did not approach toxicological limits, consistent with their nutritional roles and regulatory reference ranges.

### 3.3. Comparison with Regional and International Studies

Heavy metal concentrations measured in this study were compared with previously published data from the Marmara Sea, other Turkish coastal regions, and several international locations (Table 5). Overall, the results fall within the broad concentration ranges reported for *M. galloprovincialis* and related bivalve species globally. Zinc and copper exhibited moderately elevated concentrations compared with some Mediterranean and Aegean datasets; however, such values are consistent with previous observations indicating naturally high Zn levels in Mediterranean mussels and region-specific variability in Cu associated with local environmental conditions.

Lead concentrations in the present study were generally lower than those reported from several highly industrialized regions (e.g., Montenegrin and Albanian coasts) but slightly higher than values documented for less-impacted Turkish sites such as Yalova. Cadmium and mercury levels were comparatively low and aligned with the lower end of ranges reported for the Ionian and Tyrrhenian Seas.

It is important to note that not all studies report metal concentrations on the same measurement basis. While the current study reports values on a wet-weight (WW) basis, several international studies express concentrations as dry-weight (DW) values, which are typically 4–6 times higher than WW concentrations due to moisture loss during drying. Therefore, cross-study comparisons should be interpreted cautiously, as differences in analytical reporting, sampling season, mussel size, and environmental exposure conditions may all contribute to observed variability. Despite these methodological differences, the overall comparison suggests that heavy metal concentrations in mussels from the southern Marmara Sea are broadly comparable to those reported in other regions, with no evidence of unusually elevated contamination relative to global datasets.

### 3.4. Toxicology Results of Heavy Metals in the Mussels

Estimated Daily Intake (EDI), Estimated Weekly Intake (EWI), Target Hazard Quotient (THQ), and Hazard Index (HI) values were calculated for both adults (70 kg) and children (20 kg), recognizing that body weight substantially influences exposure and toxicological outcomes. A consumption rate (CR) of 30 g/day was applied based on the most recent Türkiye National Food Consumption Survey (TUBATUR 2017–2018) [36], which reports average seafood intake for the Turkish population. Detailed numerical values for each sampling site and population group are provided in Supplementary Table S1.

All EWI values remained below the Provisional Tolerable Weekly Intake (PTWI) limits established by FAO/WHO–JECFA, indicating no appreciable risk of chronic toxicity from weekly mussel consumption. Among the metals, Zn and Cu contributed the highest proportions to total weekly exposure; however, their EWI values (Zn: 162.5–274.9 µg/kg bw/week; Cu: 45.2–77.5 µg/kg bw/week) were still far below their respective PTWI levels (Zn: 7000 µg/kg bw/week; Cu: 3500 µg/kg bw/week). Metals such as Hg, As, and Cd contributed minimally to overall intake, reflecting their relatively low concentrations in the analyzed mussel samples.

For all stations, THQ values for individual metals remained below 1.0 for both adults and children, indicating no significant non-carcinogenic risk. Similarly, HI values (sum of THQs) were also <1.0 for all sites (adult range: 0.214–0.695; child range: 0.375–1.12). Notably, HI approached 1.0 in children at Mudanya, primarily due to elevated Zn and Cd; however, because PTWI values were not exceeded and THQs remained individually <1.0, these exposures are not expected to pose adverse health effects under current consumption patterns. Nevertheless, this result emphasizes the importance of considering vulnerable populations in seafood risk assessments.

Zinc displayed concentrations above the general food guideline (50 mg/kg); however, mussels are naturally rich in Zn, and the human body regulates Zn homeostasis efficiently through absorption–excretion mechanisms. This explains why Zn concentrations exceeded guideline limits while Zn-related THQ and EWI values remained far below toxicological thresholds. Lead slightly exceeded the EU limit (0.30 mg/kg wet weight) at some stations, and although Pb EWI and THQ values were low, the presence of Pb above regulatory levels warrants continued monitoring due to its cumulative toxicological properties. Overall, the toxicological assessment indicates that consumption of *M. galloprovincialis* from the southern Marmara Sea does not pose a significant non-carcinogenic risk for adults or children under typical consumption patterns. However, the relatively higher HI values observed in Mudanya and the elevated Pb concentrations at certain sites highlight the need for periodic monitoring and expanded multi-seasonal assessments.

### 3.5. Health Risk Assessment of Heavy Metals in the Mussels

**Figure 5:**
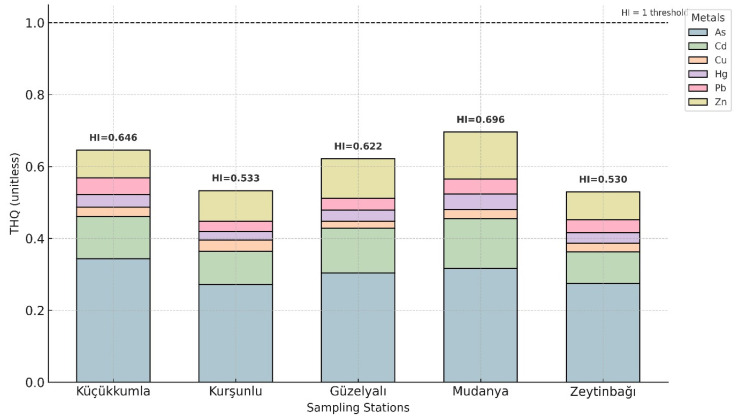
Distribution of adult THQ values for individual metals and adult total HI across sampling sites.

Non-carcinogenic health risks were estimated using Target Hazard Quotient (THQ) for individual metals and Hazard Index (HI) for cumulative exposure. THQ and HI values for adults are shown in Table 6 and Figure 5, while age-specific values for children are provided in Supplementary Table S2. Across all sampling locations, Adult THQ values for As, Cd, Cu, Hg, Pb, and Zn remained below 1.0, indicating no appreciable non-carcinogenic risk from individual metals. HI values for adults ranged from 0.529 (Zeytinbağı) to 0.695 (Mudanya), with Mudanya exhibiting the highest cumulative risk.

For the child population, Hazard Index (HI) values exceeded the threshold of 1.0 at several sampling stations, indicating a higher potential non-carcinogenic health risk compared to adults. This elevated risk is primarily attributable to lower body weight and proportionally greater dietary exposure in children.

Arsenic contributed the largest proportion to the adult HI values, followed by zinc. For the child population, THQ values for arsenic exceeded 1.0 at some stations, resulting in HI values between 1.866 and 2.461 (Supplementary Table S2). Although adult HI values remained <1, child HI values exceeded 1 at all stations, indicating a comparatively higher potential risk for children.

## 4. Discussion

The present study provides a comprehensive evaluation of heavy metal accumulation in *Mytilus galloprovincialis* from the southern Marmara Sea and integrates these findings with potential ecological and human health implications. As a filter-feeding bivalve with a high capacity for particle retention, *M. galloprovincialis* serves as an effective sentinel for coastal pollution monitoring [15,55]. The spatial heterogeneity observed in heavy metal concentrations likely reflects the interplay between localized anthropogenic inputs, maritime operations, and natural geochemical processes. However, because each sampling station was represented by a composite biological sample, spatial comparisons should be interpreted cautiously, as variability reflects analytical rather than biological replication. Despite this limitation, the observed patterns provide meaningful insight into contamination dynamics along this industrially and economically important marine corridor.

### 4.1. Spatial Contamination Patterns and Ecological Implications

Among the evaluated metals, zinc (Zn) displayed the highest concentrations, with Mudanya exhibiting markedly elevated levels relative to other stations. This pattern aligns with known urban and maritime pressures—including port operations, ship maintenance, antifouling paints, and galvanized infrastructure—which collectively contribute Zn to coastal waters [47,56]. The strong Zn–Cu correlation observed further supports the influence of marine infrastructure, as both metals commonly originate from antifouling coatings. Similar Zn enrichment has been reported in mussels from the Aegean, Adriatic, and Tyrrhenian Seas, particularly near ports and industrialized coastlines [45,46]. Although Zn is an essential micronutrient, excessive accumulation can impair enzymatic activity in bivalves, highlighting potential sublethal ecological effects.

Copper (Cu) showed its highest concentrations in Kurşunlu, a site characterized by small-scale shipyard activity and marina operations. Although measured Cu values remained well below international safety thresholds, chronic Cu exposure can induce oxidative stress, disrupt membrane functions, and impair metabolic processes in mussels [57]. This suggests that even moderate elevations may have ecological relevance, particularly in semi-enclosed basins like the Marmara Sea, where water renewal is limited.

Arsenic (As) demonstrated minimal spatial variation, suggesting a predominantly geogenic origin, likely governed by sediment–water interactions and natural mineralogical features of the basin. Consistent patterns have been noted in prior Marmara and Black Sea studies where inorganic forms of As are typically present at low yet toxicologically relevant levels [53,58]. Because inorganic As is a Group 1 carcinogen, continued monitoring remains essential despite the relatively low absolute concentrations observed.

Cadmium (Cd) levels, although below EU limits, were moderately elevated in Güzelyalı and Mudanya. Cd inputs are commonly associated with phosphate fertilizers, agricultural runoff, industrial discharges, and wastewater [59]. Similar concentration patterns were reported in mussels from the Western Mediterranean and Adriatic regions, where coastal urban influence and riverine inputs contribute to Cd enrichment [41,60]. The elevated Cd values at Mudanya may therefore reflect a combination of urban wastewater and sediment resuspension from vessel activity.

Lead (Pb) concentrations exceeded EU safety thresholds in some stations, particularly Küçükkumla. Atmospheric deposition from historical industrial emissions, urban storm water drainage, and contaminated road dust constitute dominant Pb sources in coastal ecosystems [61,62]. The relatively high Pb levels at Küçükkumla likely reflect diffuse regional inputs and ongoing legacy contamination. Although Pb concentrations remain lower than those documented for heavily industrialized coasts (e.g., Algeria, Albania), its cumulative neurotoxicity requires prioritization in future monitoring programs.

Mercury (Hg) was the least abundant metal, consistent with patterns reported in less-industrialized Mediterranean subregions [23,63]. Despite low absolute concentrations, the highest Hg values were again observed in Mudanya, suggesting contributions from local industrial activities, ship maintenance, or sediment-mediated processes. Given Hg’s strong biomagnification potential, even low concentrations warrant ongoing surveillance, particularly in species occupying higher trophic levels.

### 4.2. Source Apportionment and Supporting Evidence

Identifying the origins of heavy metals in coastal ecosystems is essential for interpreting observed spatial patterns and implementing targeted mitigation strategies. In this study, source attribution was inferred by integrating mussel bioaccumulation profiles with known regional activities and established pathways documented in previous research. Because environmental matrices such as water and sediment were not analyzed concurrently, the proposed sources should be interpreted as plausible rather than definitive, reflecting a limitation also highlighted in previous biomonitoring studies.

Table 7 provides a synthesized overview of the likely primary and secondary sources for each metal. These interpretations are supported by (i) spatial concentration gradients, (ii) correlations among specific metals (e.g., Zn–Cu), and (iii) known anthropogenic activities along the Bursa coastline. For example, the elevated Zn and Cu levels observed in Mudanya and Kurşunlu are consistent with port operations, ship maintenance facilities, and antifouling paint residues, all of which are well-recognized contributors to these metals in marine environments [56,64]. Similarly, moderate Cd enrichment in Güzelyalı and Mudanya aligns with urban wastewater influences and agricultural runoff originating from nearby catchments [60].

The general concordance between observed spatial patterns and documented regional pressures reinforces the utility of *M. galloprovincialis* as an effective tracer for both localized and diffuse pollution sources. Nonetheless, future studies incorporating multi-matrix analyses (water, sediment, suspended particulate matter) and speciation measurements would strengthen source apportionment accuracy and help disentangle overlapping anthropogenic contributions.

### 4.3. Public Health Implications and Dietary Exposure Risks

Estimated Daily Intake (EDI) and Estimated Weekly Intake (EWI) values, calculated using the nationally reported average seafood consumption rate for Türkiye, are summarized in Supplementary Table S1. Across all metals and sampling locations, EWI values remained below the corresponding Provisional Tolerable Weekly Intake (PTWI) limits established by JECFA, indicating that mussel consumption at current regional dietary patterns does not pose appreciable non-carcinogenic risk for adults. This finding is further supported by the Target Hazard Quotient (THQ) and Hazard Index (HI) values (Table 6; Figure 5), all of which were below 1.0 for the adult population, suggesting that cumulative exposure to multiple metals remains within acceptable toxicological thresholds. Although the adult population exhibited HI values below 1.0, the child-specific risk assessment revealed HI values exceeding unity at some stations, highlighting children as a more vulnerable group. This finding underscores that low risk for adults does not necessarily imply safety for younger populations with lower body mass and higher exposure per unit weight.

Arsenic and zinc were the largest contributors to the adult HI values. Although Zn is an essential micronutrient required for numerous physiological functions, high intake may interfere with copper absorption and induce gastrointestinal discomfort [42]. The Zn concentrations observed here exceeded general food guideline values but still produced THQ values < 1.0 due to the relatively modest mussel consumption rate in Türkiye. This aligns with evidence that mussels—although naturally rich in Zn—are typically consumed in small quantities compared to daily dietary zinc requirements.

Arsenic’s contribution to HI, though below PTWI limits, underscores an important consideration: the toxicological impact of As depends strongly on its chemical form. Total As measurements, as performed in this study, do not differentiate between organic (typically low toxicity) and inorganic (high toxicity, carcinogenic) species. Future research incorporating arsenic speciation would therefore improve the accuracy of risk characterization, as recommended in previous regional assessments [29,65].

Comparison with other regions shows consistency with the broader literature. Similar HI patterns—dominated by Zn and As—have been documented in mussels from the Marmara, Aegean, and Black Seas, with overall risk generally remaining low at typical consumption levels [29,65]. Nevertheless, international case studies such as those from the Albanian coastline illustrate that rapid shifts in industrial activity or untreated urban discharges can elevate Pb and Cd intake beyond PTWI thresholds [54]. This highlights the importance of continuous biomonitoring in regions experiencing dynamic coastal development, including the southern Marmara Sea.

Importantly, when exposure was evaluated for the child population—who have lower body weight and therefore higher metal intake per kilogram—the resulting THQ and HI values were substantially higher, and HI values exceeded 1.0 at all stations (ranging from 1.866 to 2.461). These findings indicate a comparatively elevated potential risk for children and reinforce the need to incorporate age-stratified exposure assessments into regulatory evaluations. Detailed values for the child population are provided in Supplementary Table S2. Overall, while adult exposure remains within safe limits under current consumption patterns, the results emphasize the need for continued monitoring, age-specific dietary risk assessment, and enhanced scrutiny of metals with high toxicological relevance, particularly Pb and inorganic As.

### 4.4. Implications for Environmental Management and Future Monitoring

The spatial patterns observed in this study indicate localized but persistent anthropogenic metal inputs, particularly in Mudanya and Küçükkumla. Although current concentrations do not pose immediate health risks for adult consumers, the ecological consequences of chronic exposure, such as oxidative stress, reduced filtration efficiency, and impaired reproductive capacity in mussels, remain important considerations for long-term ecosystem health. Sediment resuspension associated with intense maritime activity may act as a secondary source of contaminants, emphasizing the need for integrated water–sediment monitoring programs. Targeted management actions, including improved storm water control, stricter regulation of antifouling coatings, and enhanced wastewater treatment, could help mitigate identified sources. Continued biomonitoring is essential to detect temporal changes, particularly under increasing coastal development and maritime pressure, and to ensure early identification of emerging risks.

### 4.5. Overall Synthesis

Overall, the findings confirm that *M. galloprovincialis* remains a reliable bioindicator of coastal metal contamination in the southern Marmara Sea, with spatial patterns clearly reflecting localized anthropogenic pressures such as port activity, urban runoff, and maritime operations. While the measured concentrations do not suggest appreciable non-carcinogenic health risks for adult consumers under current dietary exposure scenarios, the higher HI values observed at Mudanya and Küçükkumla underscore the need for continued surveillance—particularly considering the greater vulnerability of children and other sensitive populations. Although this study provides an essential baseline for interpreting long-term trends, the absence of parallel measurements in water and sediment limits the ability to distinguish between environmental availability and organism-level accumulation processes. Addressing this gap in future research will greatly strengthen ecological risk evaluation and source apportionment. Despite these limitations, the present work offers valuable reference data for environmental management, seafood safety regulation, and regional monitoring strategies in one of Türkiye’s most socioeconomically significant marine systems. The findings highlight the importance of sustained, multi-matrix biomonitoring programs to ensure early detection of emerging risks in this dynamic coastal environment.

## 5. Conclusions

This study investigated the accumulation of heavy metals and their potential health implications in Mediterranean mussels (*Mytilus galloprovincialis*) collected from the southern coast of the Marmara Sea. This study provides an important contribution by integrating spatial contamination patterns with human health risk assessment for this region. The results revealed clear spatial variations in metal concentrations, with Zn and Hg being highest in Mudanya, Cd elevated in both Mudanya and Güzelyalı, Cu enriched in Kurşunlu, and Pb peaking in Küçükkumla, while As remained relatively uniform across sites. Health risk indicators (EDI, EWI, THQ, and HI) for all metals were below international safety thresholds, suggesting that mussel consumption from the studied areas does not pose significant non-carcinogenic risks to human health. While health risk indicators suggest no significant non-carcinogenic risk for adults, the child-specific assessment revealed HI values exceeding 1.0 at certain sites, indicating a non-negligible potential health concern for children and emphasizing the need to consider vulnerable populations in future monitoring and risk assessment frameworks. However, Zn and As were identified as the dominant contributors to total exposure, consistent with their abundance and toxicological relevance. Child-specific risk assessment revealed substantially higher THQ and HI values compared to adults, underscoring the increased vulnerability of younger populations. The comparatively higher HI levels in Mudanya and Küçükkumla further indicate localized anthropogenic inputs and emphasize the importance of focused pollution control measures. Overall, the study confirms that *M. galloprovincialis* remains a reliable bioindicator species for assessing coastal metal contamination along the southern Marmara Sea. Several limitations should be acknowledged: (i) the use of composite samples restricts biological variability assessment, (ii) the single-season sampling design limits temporal representativeness, and (iii) the absence of water or sediment metal concentrations prevents the calculation of bioaccumulation metrics (BAF/BCF). Addressing these limitations in future work will improve ecological interpretation and source attribution.

These findings emphasize the importance of sustained biomonitoring, pollution control measures, and incorporation of abiotic data, and age-stratified risk assessment approaches to safeguard public health, trace pollution sources, and promote sustainable seafood consumption and coastal ecosystem management.

## Figures and Tables

**Table 1:** Formulas and units used in metal exposure and risk assessments.

Parameter	Formula	Unit	Description/Reference Values
EDI	EDI = C × CR/BW	mg/kg/day	Estimated Daily Intake
EWI	EWI = EDI × 7	mg/kg/week	Estimated Weekly Intake
PTWI	-	mg/kg/week	Cd: 0.007, Pb: 0.025, Hg: 0.004 [34]
RfD	-	mg/kg/day	As: 0.0003, Cd: 0.001, Cu: 0.04, Hg: 0.0005, Pb: 0.004, Zn: 0.3 [36]
THQ	THQ = (EF × ED × CR × C)/(RfD × BW × AT)	unitless	Non-carcinogenic risk
HI	HI = ΣTHQᵢ	unitless	Total hazard from all metals

Parameters: C = metal concentration in mussels (mg/kg, wet weight); CR = daily seafood consumption rate (kg/day), Türkiye dietary intake survey [37]; BW = body weight (70 kg adults; 20 kg children); EF = exposure frequency (365 days/year); ED = exposure duration (30 years adults; 6 years children); RfD = oral reference dose (mg/kg·day) [36]; AT = averaging time (ED × 365 days for non-carcinogenic effects).

**Table 2:** Mean (±SD) concentrations of heavy metals (mg/kg, wet weight) in *M. galloprovincialis*.

Sampling Point	As (mg/kg)	Cd (mg/kg)	Cu (mg/kg)	Hg (mg/kg)	Pb (mg/kg)	Zn (mg/kg)
Küçükkumla	0.24 ± 0.01	0.28 ± 0.01	2.53 ± 0.12	0.042 ± 0.003	0.45 ± 0.01	55.70 ± 11.3
Kurşunlu	0.19 ± 0.05	0.22 ± 0.01	3.11 ± 0.51	0.027 ± 0.002	0.28 ± 0.01	61.32 ± 15.7
Güzelyalı	0.21 ± 0.01	0.30 ± 0.01	1.79 ± 0.32	0.037 ± 0.002	0.32 ± 0.01	78.94 ± 9.75
Mudanya	0.22 ± 0.02	0.33 ± 0.05	2.38 ± 0.26	0.053 ± 0.005	0.39 ± 0.01	94.25 ± 12.8
Zeytinbağı	0.19 ± 0.01	0.21 ± 0.01	2.28 ± 0.24	0.035 ± 0.004	0.35 ± 0.01	55.80 ± 8.35

Note: SD values represent analytical replicates of composite samples and therefore reflect analytical precision rather than biological variability.

**Table 3:** One-way ANOVA results for heavy metal concentrations in *M. galloprovincialis* among sampling sites.

Metal	Sum of Squares	df	Mean Square	F-Value	*p*-Value	Significance
As	0.0023	4	0.00058	1.12	0.384	ns
Cd	0.0312	4	0.00780	6.47	0.012	*
Cu	3.621	4	0.905	8.91	0.006	**
Hg	0.0015	4	0.00038	7.28	0.009	**
Pb	0.0458	4	0.01145	9.37	0.004	**
Zn	2491.8	4	622.95	15.42	0.001	***

Note: ns = not significant (*p* > 0.05); *: statistically significant (*p* < 0.05); **: highly significant (*p* < 0.01); ***: very highly significant (*p* < 0.001)

**Table 4:** Comparison of mean concentrations of heavy metals in mussels with international food safety standards.

Metals	This Study (mg/kg)	Threshold Value (mg/kg)	Source/Regulation	Compliance Status
As	0.22 ± 0.023	1.0	[38]	Compliant
Pb	0.37 ± 0.06	0.3	[39]	Not Compliant
Cd	0.26 ± 0.05	1.0	[39]	Compliant
Hg	0.045 ± 0.011	0.5	[39]	Compliant
Cu	2.38 ± 0.51	50.0 (food)	[40]	Compliant
Zn	63.32 ± 13.38	50.0 (food)	[40]	Not Compliant

Note: “Threshold value” refers to the maximum permissible concentration established by FAO/WHO or the European Commission for edible seafood. Compliance status indicates whether mean concentrations fall below (“Compliant”) or exceed (“Not Compliant”) the respective regulatory limits.

**Table 5:** Comparison of the results of this study and other studies (mg/kg wet weight) from different areas of the world.

Location	As	Pb	Cd	Hg	Cu	Zn	References
Ionian Sea, Italy	NE	0.86–1.7	0.31–0.64	0.05–0.23	0.7–0.77	3.85–5.76	[41]
Atlantic Coast, Morocco	NE	NE	0.4–8	NE	6.6–73.5	117–379	[42]
Tyrrhenian Sea, Italy	NE	1.67–2.49	0.32–0.49	NE	5.51–11.5	123–180	[43]
Aegean Sea, Türkiye	NE	0.49–1.72	0.04–0.52	NE	0.95–1.85	16.11–37.15	[44]
Marmara Sea, Türkiye	NE	0.219–1.15	0.296–0.74	NE	0.85–3.473	55.74–97.13	[45]
Montenegrin Coast	3.7–11.2	4.25–9.5	1.4–2.3	NE	6.25–15.25	160–221.1	[46]
Black Sea	5.5–18.3	0.01–22.92	0.09–3.32	NE	8.2–80.1	16–163	[47]
Marmara Sea	5.6–14.9	NE	NE	NE	3.9–89.4	27–243	[47]
Aegean Sea	7.2–14	NE	NE	NE	6.4–11	183–441	[47]
Algerian West Coast	NE	0.07–16.29	2.74–6.56	NE	1.39–5.28	205.4–575.9	[48]
Southeastern Black Sea	NE	0.06–5.88	0.16–0.84	NE	1.41–9.12	8.59–43.39	[49]
Adriatic Sea, Slovenia	8.3–24.9	NE	NE	0.06–0.36	4.25–7.1	60.3–155	[50]
Izmir Bay, Türkiye	1.07–2.9	0.147–0.672	0.04–0.11	0.004–0.091	0.75–9.38	17.01–127.1	[51]
Agigea Port, Romania	9.2–19.2	NE	NE	0.01–0.07	7.32–15.4	118–241	[52]
Yalova Coast, Türkiye	0.61–2.5	0.11–0.72	0.11–0.19	NE	0.21–0.99	15.93–39.66	[53]
Albanian Sea Coast	2.2–21.6	0.04–21.11	1.14–21.61	NE	2.38–61.3	79.31–1329.8	[54]
Romanian Black Sea	NE	0.001–11.02	0.006–4.69	NE	0.1–10.77	NE	[29]
Marmara Sea, Bursa Coast	0.22 ± 0.02	0.37 ± 0.06	0.26 ± 0.05	0.045 ± 0.011	2.38 ± 0.5	63.32 ± 13.38	This study

Note: All concentrations in the present study are reported on a wet-weight (WW) basis; NE: not evaluated.

**Table 6:** THQ and HI values for the adult population (70 kg) consuming *M. galloprovincialis* from the southern Marmara Sea.

Station	THQ—As	THQ—Cd	THQ—Cu	THQ—Hg	THQ—Pb	THQ—Zn	HI
Küçükkumla	0.344	0.117	0.026	0.035	0.047	0.077	0.646
Kurşunlu	0.272	0.092	0.032	0.023	0.029	0.085	0.533
Güzelyalı	0.304	0.125	0.019	0.031	0.033	0.110	0.622
Mudanya	0.317	0.138	0.025	0.044	0.041	0.131	0.695
Zeytinbağı	0.275	0.088	0.024	0.029	0.036	0.078	0.529

**Table 7:** Source attribution summary table.

Metal	Primary Source	Secondary Source	Evidence (This Study)	Literature Support
As	Geogenic	Agriculture	Minimal spatial variation; values consistent across sites	[53]
Cd	Fertilizer runoff	Sewage	Higher in Güzelyalı/Mudanya; moderate correlation with Zn	[60]
Cu	Maritime activity	Antifouling paints	High in Kurşunlu; Zn–Cu relationship supports maritime influence	[64]
Zn	Port operations	Urban runoff	Markedly elevated in Mudanya; spatial co-occurrence with Cu	[56]
Pb	Atmospheric deposition	Urban runoff	Highest at Küçükkumla; consistent with diffuse urban inputs	[61]
Hg	Port activity	Industrial runoff	Highest in Mudanya; low absolute levels but spatially distinct	[63]

## Data Availability

The original contributions presented in this study are included in the article/Supplementary Materials. Further enquiries can be directed to the corresponding author.

## Supplementary Materials

The following supporting information can be downloaded at: https://www.mdpi.com/article/10.3390/toxics13121084/s1; Table S1: EDI and EWI values of heavy metals in mussels from different sampling sites (µg/kg/day and µg/kg/week, respectively), Table S2: THQ and HI values for the child population across sampling sites, calculated using age-specific exposure parameters.
