# Supplementary material for: Heavy Metal Accumulation and Human Health Risk in Mediterranean Mussels from the Southern Marmara Sea, Türkiye
Source: Toxics. 2025 Dec 17;13(12):1084. doi: 10.3390/toxics13121084 (PMC12737311; doi:10.3390/toxics13121084)
Supplement: Supplementary file 1 [file toxics-13-01084-s001.zip › toxics-4025079-supplementary.pdf]

Table S1. Estimated Daily Intake (EDI) and Estimated Weekly Intake (EWI) values for adults (70 kg) and children (20 kg) based on a daily mussel consumption rate of 30 g (CR = 0.03 kg/day). Metal concentrations are taken from Table 2 (mg/kg, wet weight). EDI is expressed in µg/kg bw/day and EWI in µg/kg bw/week.

S1 – Küçükkumla

Metal	EDI (Adult) (µg/kg bw/day)	EDI (Child) (µg/kg bw/day)	EWI (Adult) (µg/kg bw/week)	EWI (Child) (µg/kg bw/week)
As	0.10	0.36	0.72	2.52
Cd	0.12	0.42	0.84	2.94
Cu	1.08	3.80	7.59	26.57
Hg	0.02	0.06	0.13	0.44
Pb	0.19	0.68	1.35	4.73
Zn	23.87	83.55	167.10	584.85

S2 – Kurşunlu

Metal	EDI (Adult) (µg/kg bw/day)	EDI (Child) (µg/kg bw/day)	EWI (Adult) (µg/kg bw/week)	EWI (Child) (µg/kg bw/week)
As	0.09	0.32	0.63	2.21
Cd	0.13	0.45	0.90	3.15
Cu	0.77	2.69	5.37	18.79
Hg	0.02	0.06	0.11	0.39
Pb	0.14	0.48	0.99	3.36
Zn	33.83	118.41	236.82	828.87

S3 – Güzelyalı

Metal	EDI (Adult) (µg/kg bw/day)	EDI (Child) (µg/kg bw/day)	EWI (Adult) (µg/kg bw/week)	EWI (Child) (µg/kg bw/week)
As	0.09	0.32	0.63	2.21
Cd	0.13	0.45	0.90	3.15
Cu	0.77	2.69	5.37	18.79
Hg	0.02	0.06	0.11	0.39
Pb	0.14	0.48	0.99	3.36
Zn	33.83	118.41	236.82	828.87

S4- Mudanya

Metal	EDI (Adult) (µg/kg bw/day)	EDI (Child) (µg/kg bw/day)	EWI (Adult) (µg/kg bw/week)	EWI (Child) (µg/kg bw/week)
As	0.09	0.34	0.67	2.31
Cd	0.14	0.50	0.99	3.47
Cu	1.02	3.57	7.14	24.99
Hg	0.02	0.08	0.16	0.56
Pb	0.17	0.59	1.20	4.09
Zn	40.39	141.38	282.75	989.63

S5- Zeytinbağı

Metal	EDI (Adult) (µg/kg bw/day)	EDI (Child) (µg/kg bw/day)	EWI (Adult) (µg/kg bw/week)	EWI (Child) (µg/kg bw/week)
As	0.08	0.29	0.57	2.00
Cd	0.09	0.32	0.63	2.21
Cu	0.98	3.43	6.84	23.94
Hg	0.02	0.06	0.10	0.37
Pb	0.15	0.53	1.05	3.68
Zn	23.91	83.70	167.37	585.90

Supplementary Table S2. THQ and HI values for children (20 kg body weight) consuming M. galloprovincialis from the southern Marmara Sea.

Station	THQ-As	THQ-Cd	THQ-Cu	THQ-Hg	THQ-Pb	THQ-Zn	HI (Total)
**Küçükkumla**	1.200	0.420	0.095	0.126	0.169	0.278	**2.288**
**Kurşunlu**	0.950	0.330	0.117	0.081	0.105	0.307	**1.889**
**Güzelyalı**	1.050	0.450	0.067	0.111	0.120	0.395	**2.193**
**Mudanya**	1.100	0.495	0.089	0.159	0.146	0.471	**2.461**
**Zeytinbağı**	0.950	0.315	0.085	0.105	0.131	0.279	**1.866**
